# Recurrent Abortion

**Published:** 1903-04-18

**Authors:** 


### RECURRENT ABORTION.

IN a paper published in the *British Medical Journal*, Mr. J. W. Taylor discusses the various causes which lead to repeated abortion and the treatment of the different conditions which are associated with this distressing symptom. At the outset he objects to the term "habitual abortion," for he does not believe that habit has anything to do with the causation, and that therefore the term is a misleading one. Recurrent abortion is a better term. With regard to the causes, syphilis is undoubtedly one of the most frequent, but anyone who has a large experience will be aware that in many instances there is no history or sign of syphilis and, moreover, such cases do not follow the usual course of syphilitic abortions and are not benefited by antisyphilitic treatment. If these cases be carefully investigated they will most of them be found to fall into one fairly distinct group, while the remainder belong to isolated examples which are too rarely met with to form any well-marked class. Of the rarer forms a few are due to intraperitoneal adhesions. It is the rule for such adhesions involving the uterus to stretch or give way as the uterus enlarges, but it does occasionally happen that abortion occurs when the uterus has reached a certain size. Another uncommon cause of recurrent miscarriage is chronic nephritis. In a few instances a deep laceration of the cervix may bring about repeated abortion, but more often it leads to a single abortion followed by cervicitis with eversion and hypertrophy leading to sterility. A criminal cause may receive passing mention. When all these rarer cases of recurrent miscarriage are accounted for, and when syphilis can be excluded there still remains a well defined group of cases of nearly equal importance to that which belongs to syphilis. The distinguishing features of these cases are (1) low vitality on the part of the mother or father or both parents, (2) a "strumous" family history, (3) the success which follows "anti-strumous" treatment when carried on for a long period of time. Mr. Taylor gives the results which he has obtained in 12 patients who come under this class. Their ages were all between 20 and 34, the average being 26. These patients had had on the average five miscarriages each before coming under treatment, and only two of the 12 had ever borne a living child, and in each of these instances the child died shortly after birth. Ten of the cases were treated with syrupus ferri phosphatis comp., ʒi t.d.s., and with gradually increasing doses of cod-liver oil, ʒi to ℥ss t.d.s., during the whole time they were under treatment. The other two were treated, one with cod-liver oil and one with syrupus ferri phosphatis comp. With regard to the results, five of these patients have borne healthy children, four were advanced in pregnancy beyond seven months, when the last note was taken, and three were lost sight of. Mr. Taylor has had many other similar cases where the same treatment was successfully employed, but of which cases he has no notes. In his experience the general characters of these recurrent abortions occurring in women of low vitality are different to those which are met with in syphilitic patients. In the latter cases the tendency seems to be for each succeeding miscarriage to occur at a later period of pregnancy, whereas in the other class it is likely that each successive abortion will occur at an earlier period. Again, he thinks the clinical history of the miscarriages are different. In syphilis, while abortion may occur spontaneously and without difficulty, in a large proportion the products are retained and need operative removal. In the case of patients of low vitality such an occurrence seldom occurs.

